# Activated hepatic stellate cells secrete periostin to induce stem cell-like phenotype of residual hepatocellular carcinoma cells after heat treatment

**DOI:** 10.1038/s41598-017-01177-6

**Published:** 2017-05-19

**Authors:** Rui Zhang, Rong-Rong Yao, Jing-Huan Li, Gang Dong, Min Ma, Qiong-Dan Zheng, Dong-Mei Gao, Jie-Feng Cui, Zheng-Gang Ren, Rong-Xin Chen

**Affiliations:** Liver Cancer Institute, Zhongshan Hospital, Fudan University and Key Laboratory of Carcinogenesis and Cancer Invasion, Ministry of Education, Shanghai, China

## Abstract

Some evidences show that residual tumor after thermal ablation will progress rapidly. However, its mechanisms remain unclear. Here, we assessed whether activated HSCs could regulate stem cell-like property of residual tumor after incomplete thermal ablation to promote tumor progression. Human HCC cell lines were exposed to sublethal heat treatment to simulate the peripheral zone of thermal ablation. After residual HCC cells were cultured with conditional medium (CM) from activated HSCs, parameters of the stem cell-like phenotypes were analyzed. Nude mice bearing heat-exposed residual HCC cells and HSCs were subjected to metformin treatment to thwarter tumor progression. CM from activated primary HSCs or LX-2 cells significantly induced the stem cell-like phenotypes of residual HCC cells after heat treatment. These effects were significantly abrogated by neutralizing periostin (POSTN) in the CM. POSTN regulated the stemness of heat-exposed residual HCC cells via activation of integrin β1/AKT/GSK-3β/β-catenin/TCF4/Nanog signaling pathway. Metformin significantly inhibited *in vivo* progression of heat-exposed residual HCC via suppressing POSTN secretion and decreasing cancer stem cell marker expression. Our data propose a new mechanism of activated HSCs promoting the stemness traits of residual HCC cells after incomplete thermal ablation and suggest metformin as a potential drug to reverse this process.

## Introduction

Thermal ablation, especially radiofrequency ablation (RFA), has been widely used with success to treat unresectable early hepatocellular carcinoma (HCC). For patients with small HCC, RFA can provide survival benefits similar to those achieved by radical resection^1^. Due to its minimally invasiveness and effectiveness, RFA has been increasingly expanded to treat medium-size or large HCC^2^. However, the efficacy of RFA decreases with increasing lesion size. One study has shown that complete ablation rate after RFA was 87.7% in medium (3–5 cm) HCC whereas it deceased to 57.1% in large (>5 cm) HCC. Even among the lesions of showing complete necrosis on imaging after RFA, 19.3% lesions still developed local recurrence during the follow up^3^. The residual HCC may be detected early by radiographic imaging showing an incomplete tumor response or be occult in which case an initial complete response is followed by tumor progression. Residual tumor or local tumor recurrence (starting from the previous treated lesion) is not uncommon phenomena for ablating HCC larger than 3 cm^4^ and represents the clinical challenge of hindering the therapeutic improvement. Even more importantly, emerging reports showed that rapid aggressive progression after RFA was increasingly observed^3, 5–7^, indicating insufficient thermal ablation may accelerate tumor progression. However, biological effects of incomplete thermal ablation on tumor progression are not fully explored. Controlling the progression of minimal or occult residual HCC and decreasing risk of local recurrence after thermal ablation would hopefully improve outcomes of RFA, especially for patients with large HCC nodules (>3 cm).

Given the residual tumor or local recurrence after suboptimal thermal ablation often occurring at the periphery of ablated lesions, peritumoral microenvironment may provide supports to residual HCC cells after insufficient heat treatment. Activated hepatic stellate cells (HSCs) are one of important components of the tumor microenvironment of HCC. HSCs infiltrating or surrounding HCC secrete many cytokines, chemokines, growth factors, extracellular matrix proteins to remodel tumor microenvironment^8^. Bi-directional cross-talk between activated HSCs and HCC cells promotes tumor progression by creating a favorable microenvironment^9^. In animal experiments, activated HSCs recruited at the perimeter of the necrotic zone after RFA were observed^10^. It is reasonable to hypothesize that peri-tumoral activated HSCs may provide supportive signals to residual HCC after thermal ablation.

The more aggressive behaviors of residual tumor after heat treatment have been linked with increased subpopulation of progenitor-like or liver cancer stem cells in the residual HCC^11, 12^. However, these studies focused on the changes of cancer cells in response to heat stress, neglecting the contributions of tumor microenvironment. Cancer stem cells (CSCs) have the ability of repopulating the tumor after initial treatment, but stromal niche signals are crucial to the cell fate of CSCs^13^.

Periostin (POSTN) is one of the extracellular proteins produced by activated HSCs^14^, which has been shown to promote tumor progression in other cancers^15^ including prostate cancer, breast cancer and ovarian carcinoma. Notably, POSTN contributes to the expansion of breast cancer stem cells^16^. Therefore, we hypothesize that POSTN secreted by peri-tumoral activated HSCs may induce the acquisition of stem cell-like properties in residual HCC cells after incomplete thermal ablation to promote tumor progression.

Here, we showed that (i) activated HSCs promoted stem cell-like phenotypes in residual HCC cells following sublethal heat treatment through secreting POSTN; (ii) POSTN regulated the stemness of heat-exposed residual HCC via integrin β1/AKT/GSK-3β/β-catenin/TCF4/Nanog signaling pathway; (iii) metformin, an anti-diabetic drug, inhibited progression of heat-exposed residual HCC through suppressing POSTN secretion. This study proposes a new mechanism of activated HSCs promoting the stem cell-like properties in residual HCC cells after incomplete thermal ablation through POSTN/integrin β1/AKT/GSK-3β/β-catenin/TCF4/Nanog signal pathway and metformin as a potential drug to reverse this process.

## Results

### Activated HSCs-derived CM promotes the stem-like phenotype of residual HCC cells after heat treatment *in vitro*

**Figure 1:**
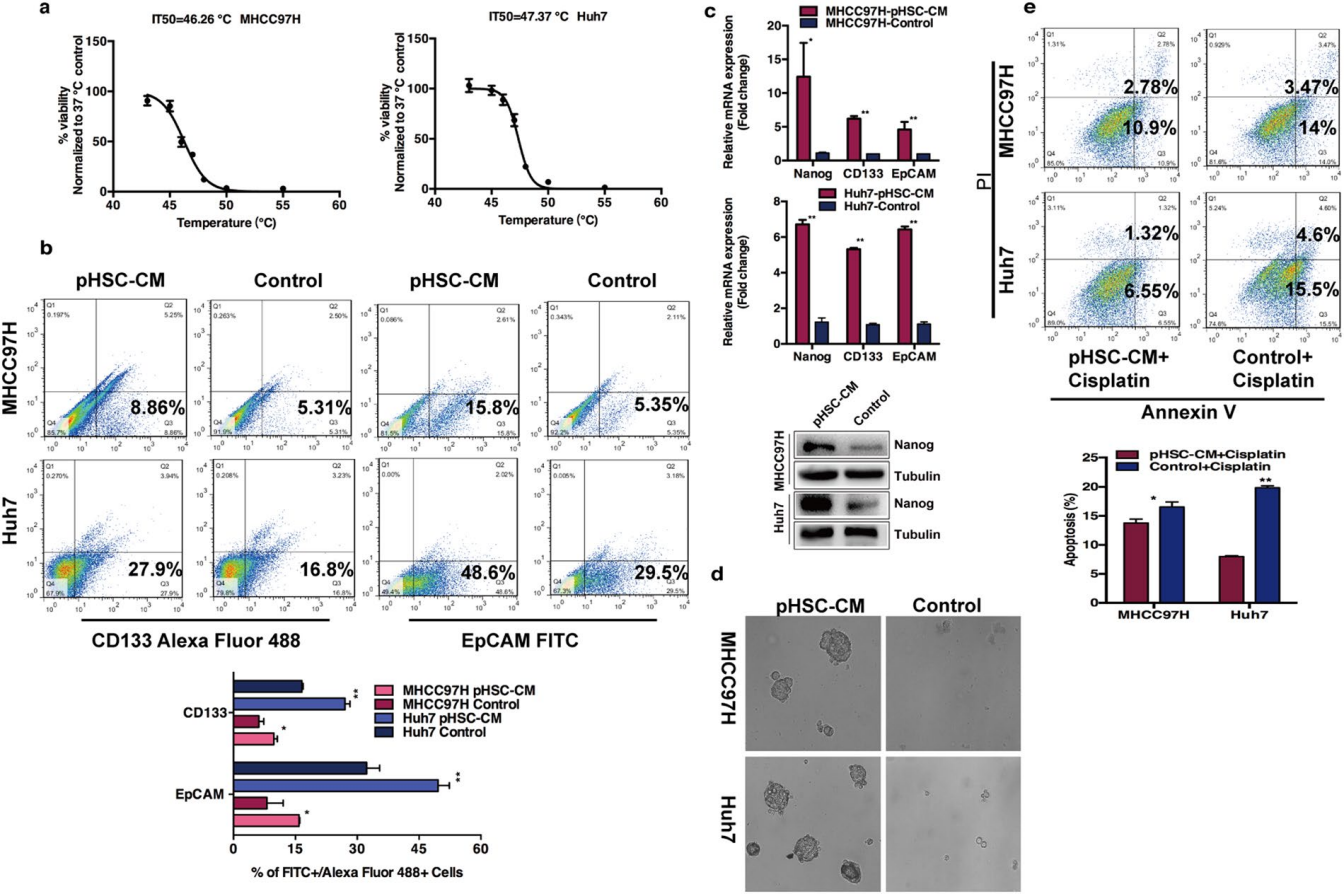
CM from activated HSCs (HSC-CM) induced the stemness traits in the residual HCC cells after *in vitro* heat treatment. (**a**) Heat stress dose-response curves of MHCC97H and Huh7 cells. IT50 indicates a temperature leading to 50% reduction of cell viability. (**b**) The proportion of CD133(+) or EpCAM(+) cells in residual MHCC97H and Huh7 cells cultured with HSC-CM or control medium were analyzed by flow cytometry. (**c**) Nanog, EpCAM and CD133 mRNA in residual MHCC97H and Huh7 cells cultured with HSC-CM or control medium were determined by quantitative RT-PCR. Western blots were performed to analyze Nanog protein expression. (**d**) Residual MHCC97H and Huh7 cells were subjected to a spheroid formation assay in the presence of pHSCs (primary human hepatic stellate cells) CM or control medium. The morphology of tumor spheroids was showed. (**e**) The apoptosis of residual MHCC97H and Huh7 cells treated with cisplatin in the presence of pHSCs-CM or control medium were evaluated by Annexin V/PI staining. **P < 0.01; *P < 0.05.

Heat stress dose-response curves of HCC cells showed IT50, a temperature resulting in 50% reduction of cell viability, was 46.26 °C for MHCC97H and 47.37 °C for Huh7 (Fig. 1a). On the basis of the IT50 data, 47.0 °C for 10 min was selected as the condition of sublethal heat treatment to recapitulate an *in vivo* incomplete thermal ablation.

After exposed to sublethal heat treatment, MHCC97H and Huh7 cells were cultured with CM from activated HSCs and subjected to analysis of the percentage cells expressing stemness markers. The percentage of EpCAM(+) or CD133(+) cells in residual HCC cells cultured with CM was significantly increased than those cultured with the control medium (Fig. 1b). Moreover, CM remarkably upregulated the expression of CD133, EpCAM and pluripotency factor Nanog in residual HCC cells after heat treatment (Fig. 1c). To determine the effects of CM on regulating the self-renewal, residual HCC cells after heat treatment were subjected to a spheroid formation assay with supplementation of either control medium or CM. When compared to cells incubated with the control medium, significantly more and larger spheroids were observed in residual MHCC97H and Huh7 cells incubated with the CM from activated HSCs (Fig. 1d). Using Annexin V/PI staining, heat-exposed residual MHCC97H and Huh7 cells treated with cisplatin showed a lower percentage of apoptotic cells when incubated with the CM of activated HSCs (Fig. 1e). The data of CM from activated HSCs inducing stem-like phenotypes of heat-exposed residual HCC cells such as enrichment of CD133(+) or EpCAM(+) cells, Nanog expression, sphere-formation ability and chemoresistance suggest that activated HSCs promote stemness of heat-treated residual HCC cells mainly through paracrine signaling.

### POSTN in the CM of activated HSCs regulates the stemness of residual HCC cells after heat treatment *in vitro*

**Figure 2:**
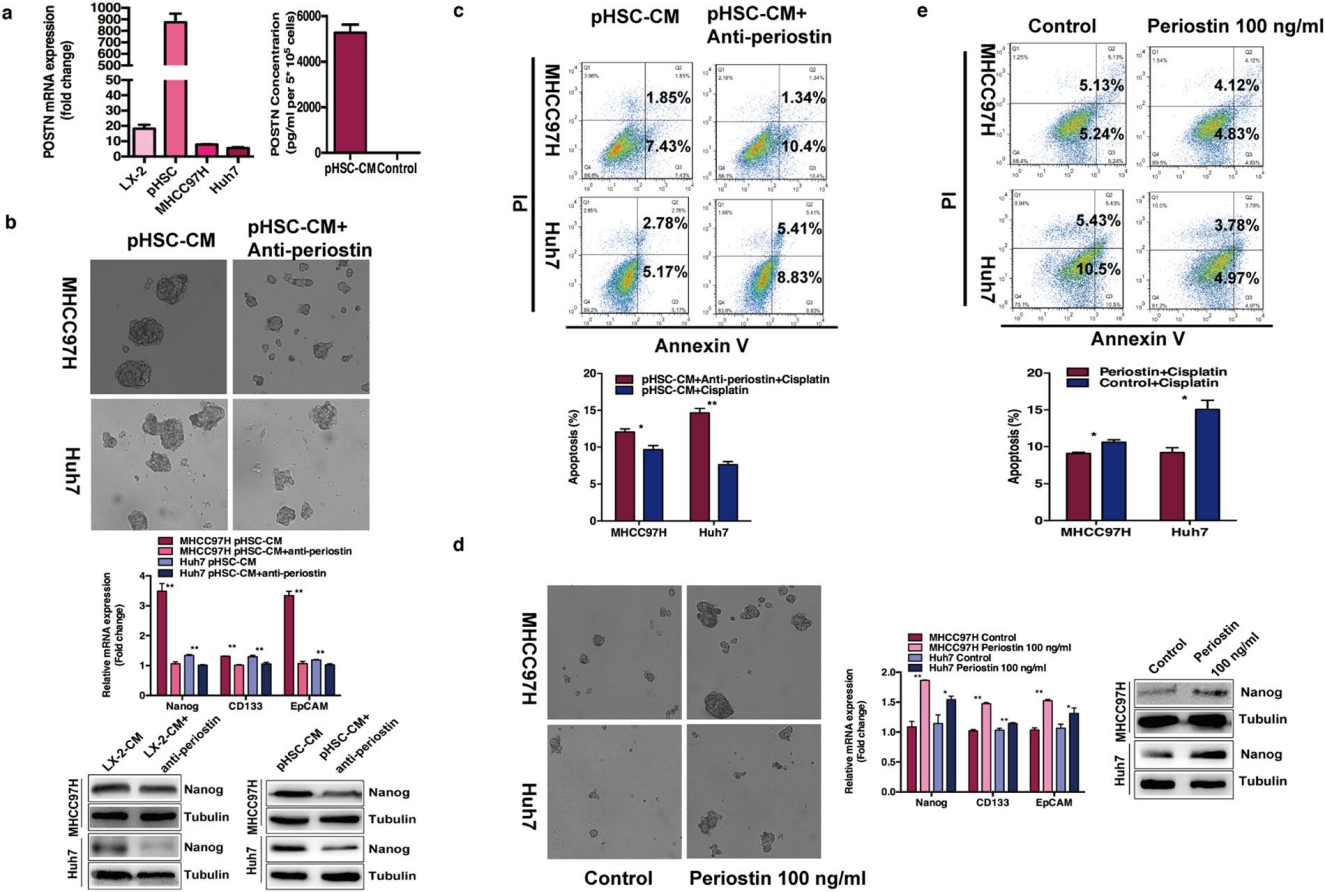
POSTN in the HSC-CM modulated the stemness of post heat-exposed residual HCC cells. (**a**) POSTN mRNA in HSC cells (LX-2, pHSCs) and HCC cells (MHCC97H, Huh7) was determined by quantitative RT-PCR. The concentration of POSTN in the HSC-CM were measured by ELISA assay. (**b**,**c**) Decreased spheroid formation ability and expression of Nanog, CD133 and EpCAM, and increased apoptosis to cisplatin in residual MHCC97H and Huh7 cells were observed after the removal of POSTN in the HSC-CM using POSTN-neutralizing antibody. (**d**,**e**) Supplement with 100 ng/ml recombinant POSTN increased the expression of Nanog, CD133 and EpCAM, and induced chemoresistances to cisplatin in the heat-exposed residual MHCC97H and Huh7 cells. **P < 0.01; *P < 0.05.

POSTN, one of secreted proteins produced by fibroblasts including activated HSCs, is involved in the enrichment of breast cancer stem cells^16, 17^. Using quantitative RT-PCR, we found HCC cell lines (MHCC97H, Huh7) produced negligible levels of POSTN while LX-2 and primary human hepatic stellate cells (pHSCs) produced a significant amount of POSTN, suggesting that activated HSCs are a major source of POSTN. Meanwhile, ELISA assay confirmed the high concentration of POSTN in the supernatant of activated HSCs (Fig. 2a). To demonstrate activated HSCs-derived CM mediating the stemness-promoting effects is due to POSTN, we assessed the functions of CM in the presence of neutralizing anti-POSTN antibody or control medium supplemented with recombinant POSTN. Upon the withdrawal of POSTN in the CM using POSTN-neutralizing antibody resulted in a decrease in the expression of Nanog, CD133, EpCAM (Fig. 2b). Spheroid formation ability of heat-exposed residual MHCC97H and Huh7 cells was reduced when the CM from activated HSCs was pre-incubated with the POSTN-neutralizing antibody (Fig. 2b). As assessed by apoptotic assay, residual MHCC97H and Huh7 cells were more sensitive to cisplatin apoptosis when cultured with CM in the presence of a POSTN-neutralizing antibody (Fig. 2c). These results showed that inhibition of POSTN activities abrogated the effect of CM of activated HSCs on the self-renewal, Nanog expression and chemoresistance in heat-treated residual HCC cells.

Recombinant POSTN exerted an effect similar to that of the CM of activated HSCs, which induced a significant increase in spheroid formation, the expression of ﻿EpCAM, CD133 and ﻿Nanog in residual MHCC97H and Huh7 cells (Fig. 2d). By Annexin V/PI staining, residual MHCC97H and Huh7 cells treated with cisplatin exhibited decrease in the percentage of the apoptotic population when cultured with control medium supplemented with recombinant POSTN (Fig. 2e). These data of supplement of POSTN phenocopying effects of activated HSCs-derived CM confirm the effects of CM on the regulation of stem-like properties in heat-treated residual HCC cells is through POSTN.

### POSTN regulates stem phenotypes of residual HCC cells after heat treatment dependent on integrin β1/AKT/GSK-3β/β-catenin/TCF4/Nanog signaling pathway

It has been reported that stromal derived POSTN promotes cancer progression including maintaining the phenotype of cancer stem cell via Akt, Wnt/β-catenin signaling pathway through binding to integrin receptors^18^. POSTN also induces proliferation and regeneration of human limbal stem cells with accompanied activation of integrin β1 and TCF4^19^. AKT/GSK-3β/β-catenin pathway has been well studied in cancer stem cell and β-catenin/TCF4/Nanog also has been reported to drive a progenitor-like cell phenotype of tumor cells^20, 21^. Therefore, we investigated whether integrin β1/AKT/GSK-3β/β-catenin/TCF4/Nanog signal pathway was implicated in POSTN promoting cancer stem phenotypes of heat-exposed residual HCC cells.

**Figure 3:**
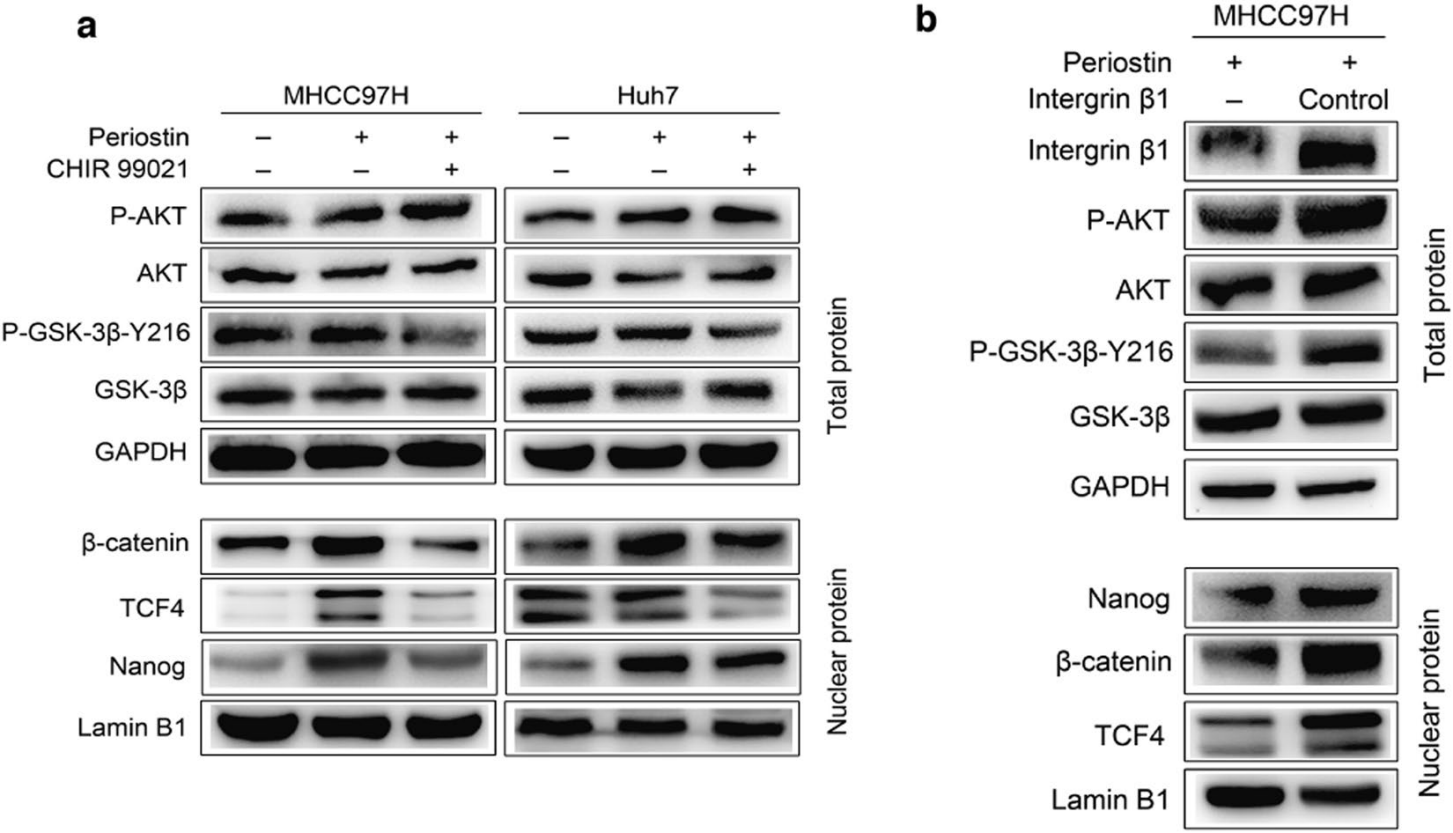
POSTN promoted the stemness of heat-exposed residual HCC cells via integrin β1/AKT/GSK-3β/β-catenin/TCF4/Nanog signaling pathway. Western blots were performed. (**a**) POSTN upregulated the phosphorylation level of AKT and GSK-3β, and the expression of nuclear β-catenin, TCF4 and Nanog in heat-exposed residual MHCC97H and Huh7 cells. The levels of GSK-3β phosphorylation, β-catenin, TCF4 and Nanog expression were reduced by the administration of GSK-3β inhibitor CHIR 99021. (**b**) Integrin β1 knockdown attenuated POSTN-induced levels of AKT and GSK-3β phosphorylation, β-catenin, TCF4 and Nanog expression in heat-exposed residual MHCC97H cells.

To define the molecular mechanism by which POSTN regulates the stemness, we treated heat-exposed residual HCC cells with POSTN. Upregulation of protein expression of Nanog in residual MHCC97H and Huh7 cells in response to POSTN treatment (Fig. 3a). Simultaneously, the phosphorylation level of AKT, GSK-3β and the expression of nuclear β-catenin and TCF4 were also upregulated (Fig. 3a). On the other hand, the GSK-3β inhibitor CHIR 99021 was used to pre-treat and then the expression of phosphorylation level of GSK-3β, β-catenin, TCF4 level and Nanog expression was reversed (Fig. 3a). Given integrins function mainly in relaying extracellular matrix signals into cell and initiating a cascade of downstream events, we used lentivirus-mediated knockdown of integrin β1 to validate whether POSTN activates the GSK-3β/β-catenin signaling pathway via integrin β1 and further influences Nanog expression. Results showed that AKT, GSK-3β, β-catenin, TCF4 levels and Nanog expression were attenuated significantly in residual integrin β1-knockdown MHCC97H cells stimulated with POSTN (Fig. 3b). Taken together, our data demonstrate that POSTN inducing stemness effects is via receptor integrin β1 and activating AKT/GSK-3β/β-catenin/TCF4 signaling pathway to regulate Nanog expression.

### Activated HSCs promote the *in vivo* progression of heat-treated residual HCC cells

**Figure 4:**
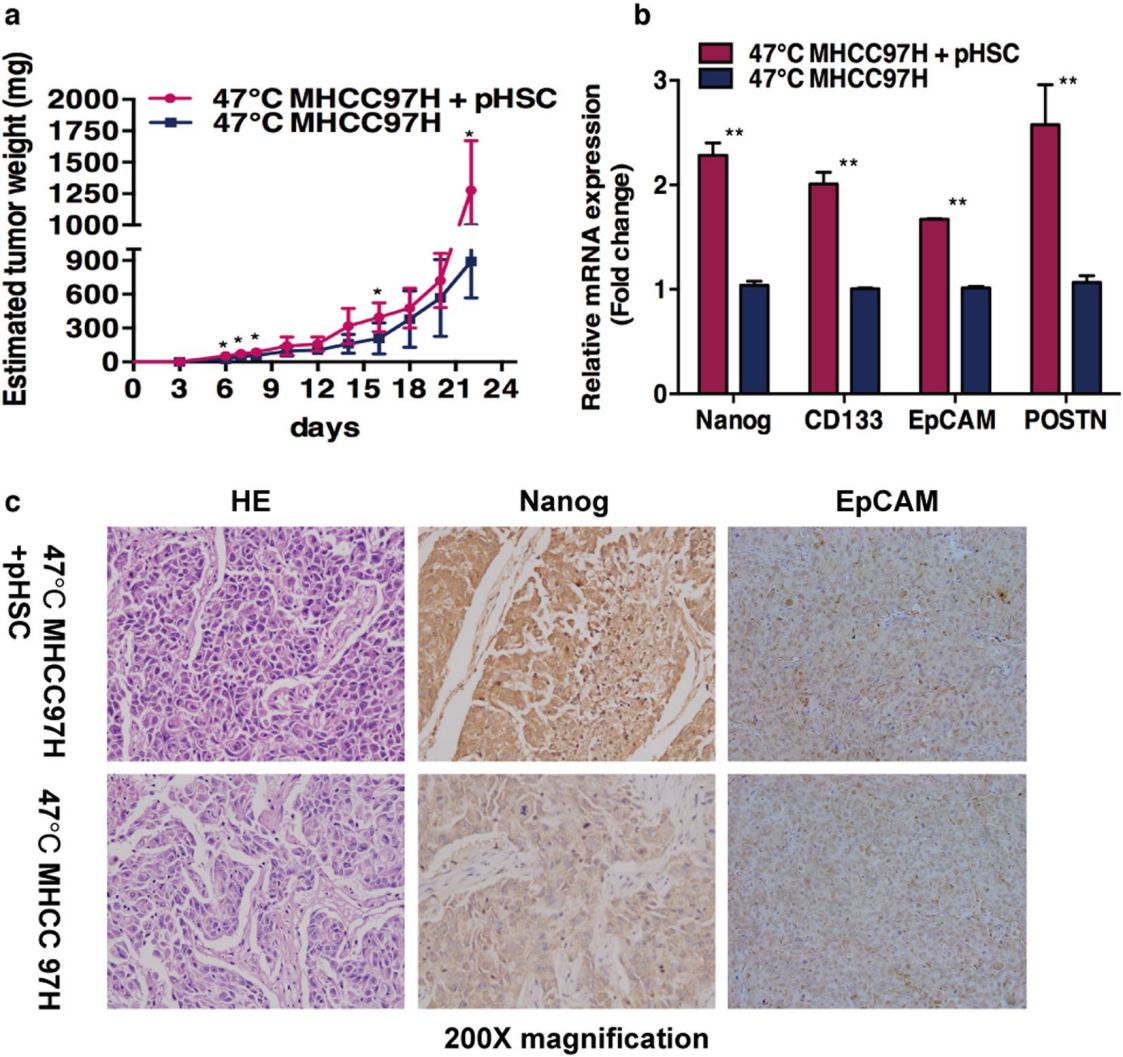
Activated HSCs promote the *in vivo* progression of heat-exposed residual HCC cells. (**a**) Heat-exposed residual MHCC97H cell co-inoculated with or without activated HSCs subcutaneously in the right flank of nude mice (n = 6 mice per group). The group of residual MHCC97H with pHSCs displayed faster tumor growth as assessed by estimated tumor weight (ETW) compared to the residual MHCC97H group. (**b**,**c**) Up-regulation of Nanog, CD133, EpCAM and POSTN in the tumors generated with residual MHCC97H with pHSCs. Quantitative RT-PCR and immunohistochemical analysis were performed. **P < 0.01; *P < 0.05.

To determine whether activated HSCs would accelerate the *in vivo* progression of residual HCC after heat treatment, we subcutaneously injected heat-exposed residual MHCC97H cells alone or with activated HSCs into nude mice. Compared to the HCC cells injected alone, residual MHCC97H cells co-inoculated with activated pHSCs exhibited greater tumor growth rate (Fig. 4a). Quantitative RT-PCR and immunohistochemical analysis showed higher POSTN, Nanog, CD133 and EpCAM expression in the group of co-inoculated of residual MHCC97H with pHSCs (Fig. 4b,c). These results show that activated HSCs promote the progression of residual HCC cells after heat treatment through POSTN secretion and enrichment of cancer stem cells.

### Metformin inhibits tumor progression of residual HCC cells via suppressing POSTN secretion from activated HSCs

It has been reported that metformin inhibits activated HSCs, fibroblasts or myofibroblasts to reverse liver, lung, cardiac or kidney fibrosis through activating adenosine monophosphate-activated protein kinase (AMPK)^22–25^.

**Figure 5:**
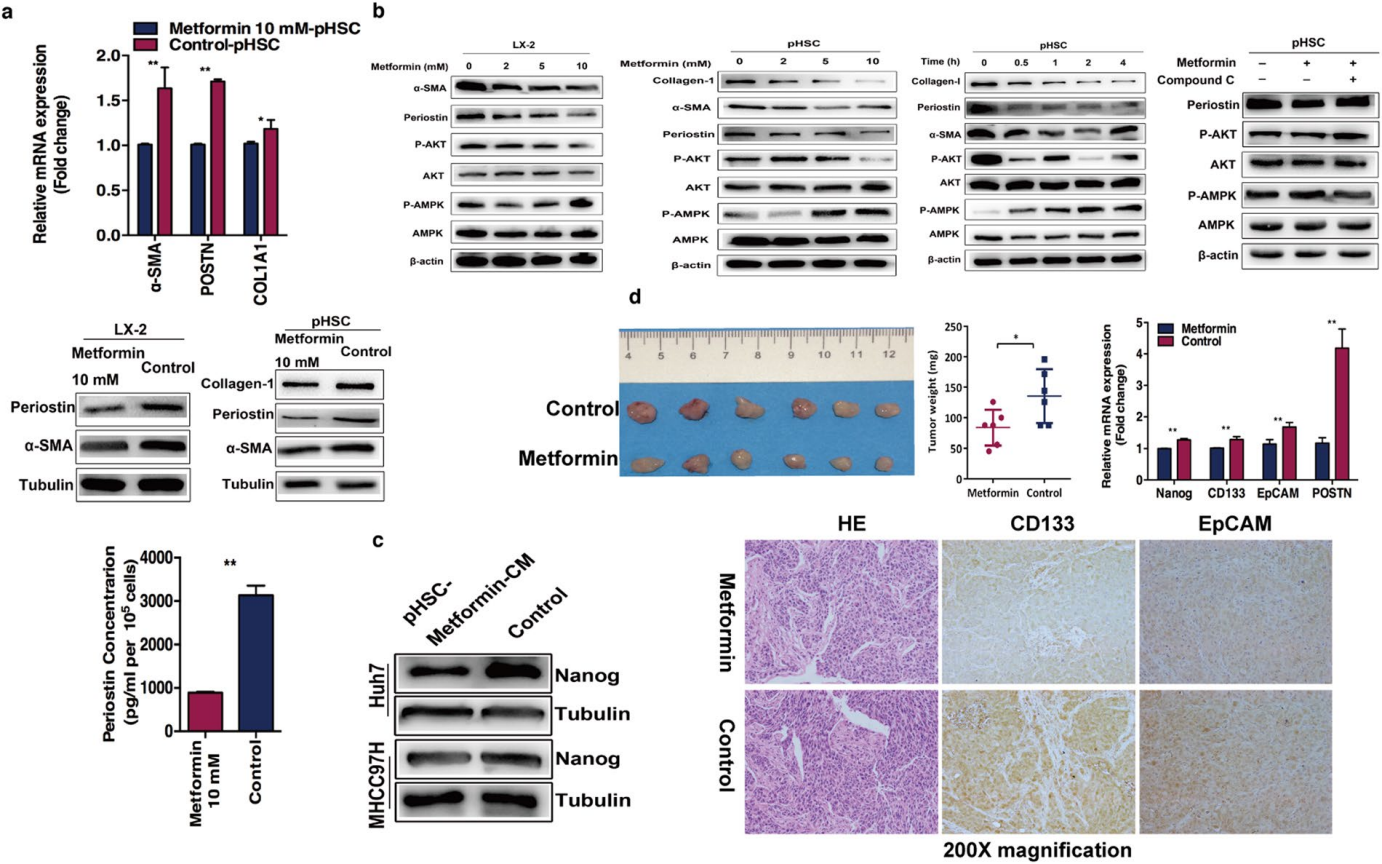
Metformin suppressed tumor progression of heat-exposed residual HCC cells by inhibiting POSTN secretion from activated HSCs and down-regulation of cancer stem cell markers. (**a**,**b**,**c**) Metformin inhibited α-SMA, collagen type 1 alpha 1 (COL1A1) and POSTN mRNA expression in the activated HSCs and decreased the supernatant concentration of POSTN in the HSC-CM. Quantitative RT-PCR and ELISA were performed. POSTN were down-regulated by metformin in dose- and time-dependent manner through p-AMPK activation and p-AKT inhibition, which was reversed by AMPK inhibitor Compound C. Western blots were performed. Nanog expression was decreased in residual MHCC97H and Huh7 cells co-cultured with CM from metformin-treated pHSCs (primary human hepatic stellate cells) compared to with control medium. (**d**) Heat-exposed residual MHCC97H cell co-inoculated with pHSCs subcutaneously in the right flank of nude mice (n = 6 mice per group). The *in vivo* tumor growth generated from heat-exposed residual HCC cells with activated HSCs was significantly inhibited in the metformin-treated group. The lower expression of Nanog, CD133, EpCAM and POSTN in the metformin-treated tumors was detected by quantitative RT-PCR and immunohistochemical analysis. **P < 0.01; *P < 0.05.

In the *in vitro* experiments, we found that metformin significantly exhibited the concentration-﻿ ﻿and time-﻿﻿dependent inhibition of HSCs activation. Except down-regulation of α-SMA and collagen type 1 alpha 1 (COL1A1), metformin treatment significantly decreased POSTN expression in the activated HSCs (Fig. 5a). Further, we found that the secretion of POSTN was reduced through activating p-AMPK and inhibiting AKT signaling after metformin treatment (Fig. 5b). AMPK inhibitor significantly reversed the inhibitory effects of metformin (Fig. 5b). These data indicate that metformin lowers POSTN expression in the activated HSCs through AMPK/AKT pathway. More importantly, the CM from metformin-treated HSCs impaired the up-regulation of Nanog expression in the residual HCC cells when compared with untreated CM (Fig. 5c).

To investigate whether metformin could inhibit *in vivo* tumor progression of residual HCC after heat treatment via inhibition of activated HSCs, we transplanted heat-exposed residual MHCC97H cells with activated pHSCs into nude mice. These were randomly divided into the untreated and metformin-treated groups. The results showed a significant inhibition of tumor growth in the metformin-treated group (Fig. 5d). Compared with untreated group, the expression of POSTN, Nanog, EpCAM and CD133 in the tumors were greatly down-regulated in metformin-treated group (Fig. 5d). These data indicate that metformin can thwart activated HSC-induced tumor progression of residual HCC cells after heat treatment through suppressing POSTN secretion and decreasing the expression of cancer stem cell markers.

## Discussion

With the more clinical use of RFA in treating medium-sized and large HCC, the increasing incidence of residual tumor or local recurrence at the ablation margin are discovered in succession, ranging from 10% to as high as 52.4%^26–28^. Even more, some studies have shown that incomplete RFA accelerates the tumor progression by inducing epithelial-mesenehymal transition or expression of the stem cell markers in residual HCC cells^11, 12^. However, whether tumor environment provides supports to residual HCC cells after thermal ablation remains unknown. In this study, we find a new mechanism that activated HSCs promotes residual HCC cells after heat treatment to acquire stem cell-like phenotypes through secreting POSTN.

Most HCC arise on a background of hepatic fibrosis or cirrhosis in which HSCs activation plays a critical role^29^. Activated HSCs have been shown to engender a tumor-permissive microenvironment to drive HCC progression^9^. Different from the changes of cancer cells themselves response to heat stress^11^, signals from peri-tumor microenvironment surrounding the ablated zone to residual tumor cannot be neglected. Like fibroblast activation playing a vital role in wound healing^30^, activated HSCs are recruited around the ablation zone after RFA^10^. Given various functions of activated HSCs^31^, it is plausible that activated HSCs provide supportive signals to residual HCC after thermal ablation to promote tumor progression.

Here, we reveal that POSTN from activated HSCs promotes stem-like characteristics in residual HCC cells after sublethal heat treatment. Cancer stem cells are considered as responsible for treatment failure to conventional therapies^32^. POSTN is one of secreted proteins produced by activated HSCs and the emerging role of the POSTN in regulating cancer stem cell has been reported in some studies^16, 17^. POSTN promotes a stem cell-like trait in breast cancer cells and contributes a microenvironmental niche supportive of the maintenance of breast cancer stem cells. Stroma-derived POSTN plays a critical role in the crosstalk between breast cancer stem cells and their niche to determine the success of metastatic colonization^16^. Cancer stem cells presence in the invasive front of nasopharyngeal carcinoma and resistance of glioma stem cells to antiangiogenic therapy are also associated with POSTN expression^33, 34^. In this study, we showed that HCC cell lines expressed low level of POSTN while activated HSCs secreted a significant amount of POSTN, and POSTN enhanced enrichment of stem-like phenotypes in heat-exposed residual HCC cells as evidenced by an increase in self-renewal, chemoresistance and the expression of stem cell markers. Neutralizing POSTN abolished the effects of CM from activated HSC on inducing stem-like phenotypes properties. These results highlight the role of activated HSCs-derived POSTN in the regulation of stem-like phenotypes in the heat-exposed residual HCC cells and suggest the therapeutic potential of targeting POSTN for treatment of residual HCC after thermal ablation.

AKT/GSK-3β/β-catenin signaling participates in the stemness maintenance of cancer cells in HCC^35^. Nanog is a canonical downstream effector of the Wnt signaling pathway^36^. In this study, the activation of integrin β1/AKT/GSK-3β/β-catenin/TCF4/Nanog pathway trigged by POSTN is identified to increase stemness in heat-treated residual HCC cells. This effect was abolished by knockdown of integrin β1 or administration of GSK inhibitor, indicating that integrin β1 and GSK-3β are the downstream effector of POSTN. POSTN supports the maintenance of breast cancer stem cells via POSTN-integrin signaling axis. Stroma-derived POSTN increases Wnt signaling in breast cancer stem cells^16^.

Furthermore, we have found that an anti-diabetic drug, metformin suppresses POSTN expression in activated HSCs through activating AMPK and inhibition of AKT activity. The emerging role of metformin in anti-fibrosis has also been reported. Metformin modulates the activity of HSCs through activation of AMPK^37^. Metformin also relieves the fibro-inflammatory microenvironment by reprogramming pancreatic stellate cells in pancreatic cancer^38^. In this study, we clearly demonstrate metformin help alleviate progression of residual HCC which is promoted by activated HSCs. POSTN expression in the activated HSCs and stem cell markers as well as tumor growth are concurrently significantly decreased after metformin treatment, indicating the therapeutic contribution from metformin through suppressing the POSTN secretion from activated HSCs and preventing the enrichment of cancer stem cells. These results suggest that metformin may be applied to inhibit progression of residual HCC and prevent local recurrence after thermal ablation.

**Figure 6:**
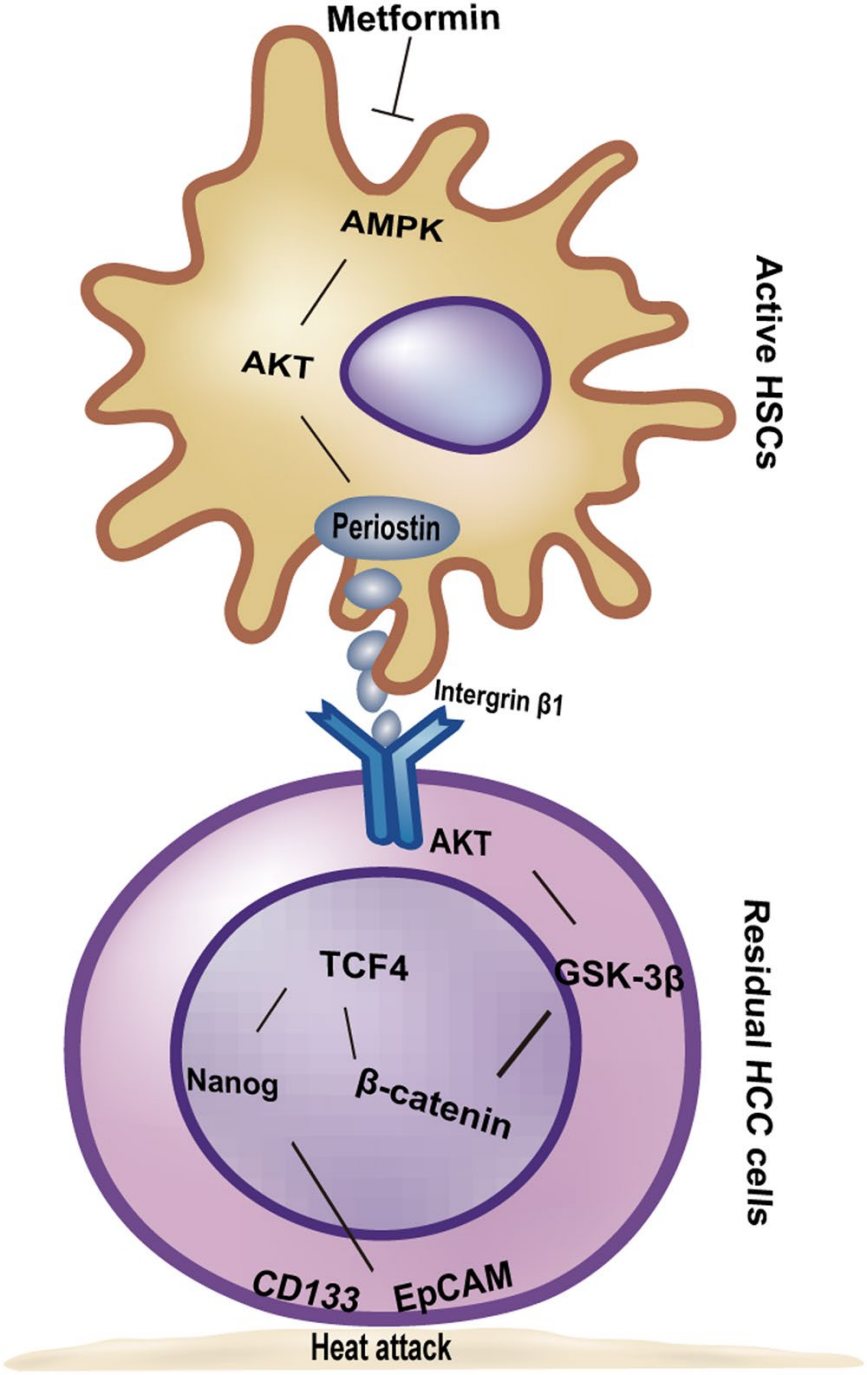
Graphical diagram of the proposed mechanism by which activated HSCs induced the stemness traits in heat-exposed residual HCC cells. POSTN from activated HSCs initiates integrin β1/GSK-3β/β-catenin/TCF4 pathway to modulate stemness traits of heat-exposed residual HCC, which was reversed by metformin via the AMPK/AKT pathway to decrease POSTN secretion.

In summary, we demonstrate that activated HSCs secrete POSTN to exert a stem-like promoting effect on the post heat-exposed residual HCC cells, which could be targeted by metformin (Fig. 6). Our study provides a better understanding of how tumor microenvironment promotes progression of residual HCC after thermal ablation and reveals a potential target that could be used for the development of combined therapeutic strategies for inhibiting and preventing residual HCC and local tumor recurrence after thermal ablation.

## Methods

### Cell culture

HCC cell lines, MHCC97H (established by Liver Cancer Institute, Fudan University, Shanghai, China), MHCC97H with integrin β1 knockdown (a gift from JF Cui) and Huh7 (obtained from the Japanese Cancer Research Bank) were cultured in Dulbecco’s modified Eagle’s medium (DMEM) with 10% fetal bovine serum (FBS, Gibco) and 1% penicillin-streptomycin (Invitrogen). The stellate cell line LX-2 cells (a gift from S. Friedman, Mount Sinai, New York) were grown in DMEM containing 2% FBS. Primary human hepatic stellate Cells (pHSCs) (ScienCell, USA) were maintained in the complete stellate cell medium. All cell cultures were conducted at 37 °C in a humidified atmosphere of 5% CO_2_.

### Heat treatment *in vitro*

When HCC cells were grown to 70% confluence, cells were harvested by trypsinized, washed and re-suspended with 1 mL of DMEM with 10% FBS in 1.5 mL micro-centrifuge tubes (5 × 10^4^ cells), and immediately exposed to heat stress in an iso-thermic water bath incubator (Jing Hong Laboratory Instrument Co., Ltd., Shanghai, China) set to the target temperature of 37 °C, 43 °C, 45 °C, 46 °C, 47 °C, 48 °C, 50 °C, and 55 °C for 10 min. Cells were then seeded into 96-well culture plates (1 × 10^4^ cells per well) in 100 μl DMEM with 10% FBS and maintained at 37 °C for 48 h. Culture medium was exchanged three times per day to remove dead cells and debris. At 48 h after heat stress, absorbance was measured to indicate cell viability using WST-1 assay (Roche) according to the manufacturer’s instructions. Absorbance was measured on a multiskan spectrum reader (Thermo Scientific). IT50, the temperature inducing a 50% reduction in cell viability relative to 37 °C control, was calculated using nonlinear regression curve fitting by Prism 6.0 (GraphPad Software). The IT50 data were used for subsequent experiments to simulate *in vivo* sublethal heat stress condition.

### Conditioned media (CM)

When LX-2 or pHSCs cells were grown to 80% confluence in 25-cm^2^ flasks, cells were washed with PBS, the medium was exchanged with fresh culture medium and incubated for 24 h. The CM of HSCs was collected and preserved in −80 °C, followed by centrifugation with 3000 rpm for 15 min to remove debris. Culture medium without cells incubated for 24 h in culture flasks served as the control.

For neutralizing POSTN in the CM of HSCs, CM was pre-incubated with anti-human POSTN antibody (2,500 ng/ml) (Abcam) or its control antibody for 1 h at room temperature before it was subjected to subsequent experimental use.

For individual experiments, HSCs were exposed to 10 mM metformin (Sigma-Aldrich) or DMSO for 12 h, then the medium was discarded and replaced with fresh medium. After 24 h, CM from metformin-treated HSCs was collected for subsequent experiments.

### Quantitative reverse transcriptase PCR (RT-PCR)

Gene expression was measured using quantitative RT-PCR as previously described^39^. In brief, total RNA was extracted using TRIzol reagent (Ambion). Then the cDNA synthesis reaction was carried out with 2 μg of total RNA using RevertAid First Strand cDNA Synthesis Kit (Thermo Scientific). Subsequently, gene expression was analyzed with Maxinma SYBR Green qPCR Master Mix (Thermo Scientific). Quantitative analysis of PCR data was performed with the 2^−ΔΔCt^ method using glyceraldehyde-3-phosphate dehydrogenase (GAPDH) or β-actin Ct values for normalization. Melting analysis was conducted to check the specificity of PCR products. The primers used in the PCR are listed in Supplementary Table S1.

### Western blot

The western blot procedures were performed as the previously described^40^. Briefly, total proteins were extracted using RIPA (Radio-Immunoprecipitation Assay) Lysis Buffer containing 1 mM PMSF (Phenylmethanesulfonyl fluoride) (Beyotime, Beyotime Institute of Biotechnology, Shanghai, China) and 10% PhosSTOP phosphatase inhibitor Cocktail (Roche) and subjected to 10% SDS-PAGE. The equal amounts of separated proteins (20 μg) were transferred onto PVDF membranes (Millipore, USA) and incubated with primary antibodies against POSTN (1:1000, Abcam), collagen I (1:1000, Abcam), α-SMA (1:300, Abcam), AMPK α (1:1000, CST, Cell Signal Technology), p-AMPK α (1:1000, CST), AKT (1:1000, CST), p-AKT (Ser 473) (1:2000, CST), GSK-3β (1:1000, CST), p-GSK-3β (1:1000, Tyr 216) (Abcam), integrin β1(1:1000, CST), TCF4 (1:1000, CST), β-catenin (1:1000, CST), Nanog (1:2000, CST), β-actin (1:1000, Beyotime), Tubulin (1:1000, Beyotime), Lamin B1 (1:1000, InTech) and corresponding HRP-conjugated secondary antibodies (Jackson). Bands were detected using Ncm-ECL Ultra (New Cell & Molecular Biotech Co., Ltd, China).

### Spheroid forming assay

Cells that can survive anchorage-independent conditions form spheroids whereas cells that cannot survive die or are loosely floating. Following exposed to sublethal heat stress (47 °C for 10 min), HCC cells were seeded into ultra-low attachment 6-well plates (2 × 10^4^ cells per well) (Corning Inc. PA, USA) and cultured in complete DMEM/F12 supplemented with 50% (v/v) CM or 100 ng/ml recombinant human POSTN (R&D Systems, Wiesbaden, Germany). The cells were photographed using phase contrast microscope (Leica, Germany). Experiments were carried out in triplicate.

### Flow cytometry (FCM) analysis

CD133 Alexa Fluor 488-conjugated Antibody (Novus Biologicals) and EpCAM FITC Conjugated Antibody (Absin Bioscience Inc.) were used to detect the proportion of positive HCC cells. Following sublethal heat stress, HCC cells (1 × 10^6^ cells/ml) were seeded into 6-well plate, incubated with HSC-CM for 48 h. The cells were collected, washed with stain buffer (BD Biosciences), re-suspended in 100 μl stain buffer containing CD133 or EpCAM antibody for 30 min at 4 °C, respectively. Followed by washing in stain buffer and incubating with secondary antibodies for 30 min, FCM was used for analysis using an excitation wavelength at 488 nm filter for fluorescent detection.

After exposure to sublethal heat stress, HCC cells were plated into 6-well plate, incubated with 100 ng/ml POSTN for 48 h and followed by the treatment of 3.3 μg/ml cisplatin for another 24 h. For analysis of apoptosis using Alexa Fluor 488 Annexin V Kit (Invitrogen), cells (1 × 10^6^ cells/ml) were harvested, washed with PBS and centrifuged at 1000 rpm for 5 min. Subsequently, cell pellets were re-suspended in annexin-binding buffer, incubated with annexin V and PI working solution for 15 min at room temperature. Cell apoptosis was measured using FACS caliber Flow cytometer (BD Biosciences, San Jose, CA, USA) and FlowJo software (Tree Star, San Carlos, CA).

### GSK-3β kinase blocking assay

After heat treatment, HCC cells were seeded in 6-well plates and incubated with fresh medium for 24 h. The cells were pre-treated with 10 μM GSK-3β inhibitor CHIR 99021 (Cayman, USA) for 4 h, followed by the treatment of 100 ng/ml POSTN for another 2 h or 24 h. Then, cells grown to 80% confluence were collected for further analysis.

### AMPK kinase blocking assay

HSCs were plated on 6-well plates overnight and pre-incubated with AMPK inhibitor, 10 μM compound C (Selleck Chemicals, China) for 3 h prior to treatment with 10 mM metformin for another 4 h or 24 h. Subsequently, cells grown to 80% confluence were collected and subjected to western blot analysis.

### Enzyme-linked immunosorbent assay (ELISA)

The concentrations of POSTN in the cell supernatant of HSCs were quantified using ELISA kit (R&D Systems, Wiesbaden, Germany) according to manufacturer’s instructions.

### Immunohistochemical staining

Immunohistochemical staining was performed as previously described^41^. Tumor specimens were removed, placed in 4% paraformaldehyde, and sliced into 5 mm thick sections. Sections were incubated with primary antibodies against Nanog (1:100, Abcam), POSTN (1:100, Abcam), CD133 (1:50, Novus), EpCAM (1:100, Abcam) overnight followed by EnVisionTM two-step Visualization System (GeneTech, Shanghai, China). Finally, slides were counterstained with Mayer’s hematoxylin and covered with coverslips. Photographs under a light microscope under ×200 magnification at 5 random fields were randomly captured with identical parameters.

### Animal experiment

All animal experimental protocols were approved by the Ethical Committee on Animal Experiments of Animal Care Committee of Fudan University, Shanghai, China. All animal experiments were carried out in accordance with the guidelines established by the Shanghai Medical Experimental Animal Care Commission. Mice were maintained under specific pathogen-free conditions and all efforts were made to minimize animal suffering. All mice were purchased from SLAC Laboratory Animal Co., Ltd., Shanghai, China.

Male BALB/c nu/nu mice weighing 18–20 g at 4–6 weeks of age were obtained. Mice were injected subcutaneously with a cell suspension containing 2 × 10^7^ heat-treated residual HCC cells alone (n = 6) or with 5 × 10^6^ pHSCs (4:1) (n = 6) in the upper right flank of each mouse. The experiment was continued for 24 days. Tumor growth kinetics was recorded every 2 days after injection. Estimated tumor weight (ETW) was measured with vernier caliper and calculated with ETW = length (mm) × (width (mm))^2^/2.

Another 12 mice bearing tumors generated from heat-treated residual HCC cells with pHSCs were randomly divided into two groups: intraperitoneal injected with either 0.9% sodium chloride (control group; n = 6) and metformin treatment (250 mg/kg/day, dissolved in 0.9% sodium chloride, metformin group; n = 6). The treatment was continued for 2 weeks. Animals were sacrificed 48 h after the last treatment and tumors were removed, weighed and fixed with 10% formalin or frozen in liquid nitrogen.

### Statistical Analysis

Data were expressed as means ± standard deviation. All statistical analyses were performed using the GraphPad Prism Software (GraphPad Software, San Diego, CA). Student’s *t* test was used for continuous data wherever appropriate. A *P*-values less than 0.05 was considered statistically significant.

## Electronic supplementary material

Supplementary Table S1
