# Supplementary material for: Activated hepatic stellate cells secrete periostin to induce stem cell-like phenotype of residual hepatocellular carcinoma cells after heat treatment
Source: Sci Rep. 2017 May 19;7:2164. doi: 10.1038/s41598-017-01177-6 (PMC5438402; doi:10.1038/s41598-017-01177-6)
Supplement: Supplementary file 1 — Supplementary Table S1 [file 41598_2017_1177_MOESM1_ESM.pdf]

# Activated hepatic stellate cells secrete periostin to induce stem cell-like phenotype of residual hepatocellular carcinoma cells after heat treatment

Rui Zhang, Rong-Rong Yao, Jing-Huan Li, Gang Dong, Min Ma, Qiong-Dan Zheng, Dong-Mei Gao, Jie-Feng Cui, Zheng-Gang Ren, Rong-Xin Chen

**Table S1** Primers for quantitative RT-PCR

| Gene symbol | Primer |
|---|---|
| POSTN | Forward 5'-GACCGTGTGCTTACACAAATTG-3' |
| | Reverse 5'-AAGTGACCGTCTCTTCCAAGG-3' |
| Nanog | Forward 5'-AGGCAAACAACCCACTTCTG-3' |
| | Reverse 5'-TCTGCTGGAGGCTGAGGTAT-3' |
| CD133 | Forward 5'-GCGATCAAGGAGACCAAAGA-3' |
| | Reverse 5'-GACCGCAGGCTAGTTTTCAC-3' |
| EpCAM | Forward 5'-ATCCTGACTGCGATGAGAGC-3' |
| | Reverse 5'-TGTCCTTGTCTGTTCTTCTGACC-3' |
| COL1A1 | Forward 5'-AGAGGAAGGAAAGCGAGGAG-3' |
| | Reverse 5'-GGACCAGCAACACCATCTG-3' |

| | |
|---|---|
| α-SMA | Forward 5'-ATGTGCGACGAAGACGAGA-3' |
| | Reverse 5'- TTCTGACCCATACCGACCAT-3' |
| GAPDH | Forward-5'-GGAGCGAGATCCCTCCAAAAT-3' |
| | Reverse-5'-GGCTGTTGTCATACTTCTCATGG-3' |
| β-actin | Forward 5'-CATGTACGTTGCTATCCAGGC-3' |
| | Reverse 5'-CTCCTTAATGTCACGCACGAT-3' |
